# Development of a health index for stranded marine tetrapods

**DOI:** 10.1371/journal.pone.0319250

**Published:** 2025-03-31

**Authors:** André S. Barreto, Karla R. K. Andrioli, Ana Paula F. R. L. Bracarense, Marta J. Cremer, Daphne W. Goldberg, Guilherme Guerra-Neto

**Affiliations:** 1 LIBGeo, Escola Politécnica, UNIVALI, Itajaí, Santa Catarina, Brazil; 2 Programa de Pós-Graduação em Ciência e Tecnologia Ambiental, UNIVALI, Itajaí, Santa Catarina, Brazil; 3 Laboratory of Animal Pathology, Universidade Estadual de Londrina, Londrina, Paraná, Brazil; 4 Laboratório de Ecologia e Conservação de Tetrápodes Marinhos e Costeiros – TetraMar, Universidade da Região de Joinville – UNIVILLE, São Francisco do Sul, Santa Catarina, Brazil; 5 Instituto Albatroz, Cabo Frio, Rio de Janeiro, Brazil; 6 Zoobotânico de São José do Rio Preto, São José do Rio Preto, São Paulo, Brazil; MARE – Marine and Environmental Sciences Centre, PORTUGAL

## Abstract

This study presents the development and validation of a Health Index (HI) to assess the overall health of stranded marine tetrapods (seabirds, sea turtles, and marine mammals). The HI is based on parameters collected during necropsies and histopathological analyses, including body condition, systemic conditions (macroscopic and by histopathology), cutaneous injuries, organized lesions, parasitosis, lymphoid depletion, and thyroid alterations. The HI was applied to data from 6,332 marine tetrapods stranded along the Brazilian coast. Results showed that the HI effectively differentiated between animals with good, fair, and poor health, as evaluated by independent veterinary assessments. Sea turtles exhibited the lowest average HI, suggesting poorer health conditions compared to seabirds and marine mammals. The HI has proven to be a valuable tool for monitoring the health of marine tetrapod populations. Using a quantitative measure of an animal’s health enables the evaluation of spatial and temporal variations in health status and the identification of more subtle impacts on species or populations. Thus, the HI allows for an objective assessment and comparison of wildlife health, supporting conservation efforts and helping to identify potential threats.

## Introduction

The integrity of an ecological system is often reflected in the health of its organisms [1]. As the effects of climate change and environmental degradation have been better characterized, concerns about the health of aquatic ecosystems have increased [2,3]. Aquatic animals may be susceptible to a wide range of emerging diseases, potentially changing the health of individual animals and their populations, and thus reducing the medium- and long-term animal survival, reproduction, and growth, therefore compromising their resilience to changes in the environment [4–7].

The effects of exposure to multiple stressors on the marine environment can be seen at multiple trophic levels of an ecosystem. In this respect, large marine vertebrates can be extremely valuable to integrate environmental quality over wide areas when there is a sufficient understanding of their distribution and behavior [8]. Organisms with long lifespans allow the study of chronic effects of stressors, including reproductive changes, abnormalities in growth and development, and tumoral growths [9–11].

There is a consensus that human health is interconnected with the health of animals and the environment [12]. Therefore, identifying changes in wildlife health is crucial. Marine tetrapods, which include mammals, reptiles, and seabirds, are key components of marine ecosystems, directly contributing to the performance of ecosystem services essential to quality of life. These species are considered environmental sentinels, being a natural component of the “One Ocean - One Health” paradigm, alerting us to potential and emerging impacts affecting these ecosystems [11,13]. Therefore, large marine vertebrates can act as “sentinels” of anthropogenic, biotic, or abiotic environmental stressors, providing warnings about negative impacts on marine ecosystems. This allows for better characterization and management of impacts that may ultimately affect both animal and human health [11,13,14]. Many analytical approaches have been employed to assess ocean health and the impacts of stress on marine vertebrate populations. Applying protocols or indexes based on data from clinical examinations and biochemical, immunological, and microbiological techniques, combined with pathological examinations and behavioral analyses, has led to the development of health assessment methods at individual and population levels. With these results, it was possible to understand the relationship between exposure to environmental stressors and a series of disease outcomes in sentinel species as an indicator of ecosystem health and animal welfare [15–17].

Since 2015, a large-scale beach monitoring program has been surveying more than 2000km of the Brazilian coastline: The Santos Basin Beach Monitoring Project (*Projeto de Monitoramento de Praias da Bacia de Santos* - PMP-BS). This project is part of the federal environmental licensing conducted by the Brazilian Institute for the Environment and Renewable Natural Resources (IBAMA) for PETROBRAS, the largest oil company in the area. The program aims to assess the potential impacts of oil and natural gas production and transport in the Santos Basin on seabirds, sea turtles, and marine mammals. Its primary activities include regularly monitoring all beaches in the area, providing veterinary care to debilitated live animals, and performing necropsies on deceased animals to determine the causes of death.

During the PMP-BS’ first five years of activity, more than 128,000 individuals were recorded along beaches and almost 23,000 necropsies were performed. As many variables were collected for each specimen, the integration of this information has been a challenge. A comprehensive approach is essential to understand the potential impacts of the human activities, specifically the industry under consideration, as oil and gas exploration can generate sub-lethal effects, chronic impacts and thus alter an individual’s health. For this reason, a quantitative health evaluation was developed, the “Health Index” (HI). The HI aims at integrating different data gathered during necropsies and auxiliary analyses, allowing for an objective, population-level evaluation of animal health. This work reports on the HI itself and its use to understand the population health of several tetrapod species recorded at the Santos Basin. It is expected that this index can be used as a proxy for the long-term monitoring of population health, based on the analysis of dead animals found on beaches.

## Methods

### Conceptual approach

Health assessment was deemed crucial for the PMP-BS’ objectives, because diagnosing the cause of death alone was insufficient for monitoring chronic impacts. Although there is an intuitive knowledge about the concept of health, a specific and standardized criterion was needed to respond to the project’s objective. Thus, the “Health Index” (HI) was proposed, considering biological, morphological, and macroscopic aspects collected during the necropsy and subsequent histopathological analyses.

When developing the HI, some assumptions were considered:

1. The index should apply to all marine tetrapods (seabirds, sea turtles, and marine mammals) usually collected during beach monitoring.
2. The index must be based on objective and defensible criteria.
3. The variability exhibited by the index should help assess the general health of a population.
4. The parameters assessed by the index should usually be evaluated during a standard necropsy and histopathological examination.

### Health index parameters

Considering the previous assumptions, eight parameters were selected, four of which were collected during the macroscopic examination (body condition; systemic conditions; cutaneous injuries; organized lesions) and four originating from histopathological analyses (systemic conditions; parasitosis associated with histopathological changes; lymphoid depletion; thyroid alterations).

The HI value is calculated by assigning a score to each parameter, which varies depending on the analyzed organ condition. As lesions in different organs have different impacts on an animal’s health, the value also considers the affected system/organ using weights to multiply the base value. The higher the parameter’s value, the greater the severity, and the worse the animal’s health condition. The description of each parameter and the criteria for their evaluation are as follows:

1. **Body score** – subjective parameter related to the animal’s body condition defined in the external examination during the necropsy.

Score: 0 – excellent; 1 – good; 2 – thin; 3 - cachectic.

Parameter Weight: This is the only parameter with different weights for seabirds and mammals (weight 1) compared to sea turtles (weight 2). The faster metabolism of endotherms (birds and mammals) causes the body condition to change much faster than that of ectotherms (reptiles). Thus, it was considered that the body score should have a higher weight in the HI for the latter.

1. **Systemic conditions (macroscopy)** - parameter related to the number of organ systems affected by pathologies identified during necropsy (macroscopic assessment).

Score: 0 - no affected systems; 1 - 1 to 3 affected systems; 2 - 4 to 6 affected systems; 3 - more than 6 affected systems.

Parameter weight: Even though all organic systems are essential for the survival of an animal, the weight attributed to this parameter varies depending on which system is affected, considering each system’s importance for maintaining the animal’s homeostasis in the short term: 1 – reproductive, musculoskeletal, and lympho-hematopoietic systems; 2 – digestive, respiratory, and urinary systems; 3 – nervous, endocrine, and cardiovascular systems.

1. **Cutaneous injuries** - This parameter relates to the distribution and intensity of injuries affecting the skin and subcutaneous tissue that are not related to anthropogenic interaction (e.g., ulcers, injuries caused by ectoparasites, dermatitis, and dermatophytosis).

Score: 0 – no lesion, 1 - focal lesions, 2 - multifocal lesions, 3 - diffuse lesions (affecting at least 60% of the body surface area).

Parameter weight: 1

1. **Organized injuries**—This parameter pertains to the presence of old and resolved injuries, such as consolidated fractures, areas of fibrosis, and cirrhosis. These injuries are typically identified during necropsies, although histopathological results may be needed for confirmation. Score: 0 - no affected systems; 1 - 1 to 3 affected systems; 2 - 4 to 6 affected systems; 3 - more than 6 affected systems.

Parameter weight: 1.

1. **Systemic conditions (histopathology)**— Similar to parameter 2, this parameter relates to the number of systems affected by pathologies, but it is based on evaluations performed during histopathological analysis.

Score: 0 - no affected systems; 1 - 1 to 3 affected systems; 2 - 4 to 6 affected systems; 3 - more than 6 affected systems.

Weight: Similar to parameter 2, systemic conditions reflect the number of organ systems affected by pathologies, based on histopathological analysis. 1—reproductive, musculoskeletal, and lympho-hematopoietic systems; 2—digestive, respiratory, and urinary systems; 3—nervous, endocrine, and cardiovascular systems.

1. **Parasitosis associated with histopathological alterations** – as parasites are expected in wildlife, a value is assigned to this parameter only if parasites are associated with tissue lesions, as evidenced by histopathological analysis.

Score: 0 - without parasite lesions; 1 - parasitosis associated with mild injury; 2 - parasitosis associated with moderate injury; 3 - parasitosis associated with severe injury.

Parameter weight: 1

1. **Lymphoid depletion** - This parameter considers the lymphocyte loss in lymphoid organs.

Score: 0 - no depletion; 1 - mild depletion; 2 - moderate depletion; 3 - severe depletion

Parameter weight: 2.

1. **Thyroid alterations**— This parameter indicates changes in the thyroid gland, such as inflammation, hemorrhage, degeneration, necrosis, atrophy, hyperplasia, among others.

Score: 0 - no change; 1 - mild change; 2 - moderate change; 3 - severe change

Parameter weight: 2.

For parameters that consider organ systems, we used those outlined in standard necropsy protocols (e.g., [18],): integumentary, cardiovascular, nervous, respiratory, digestive, urinary, lympho-hematopoietic, endocrine, musculoskeletal, and reproductive.

When assessing parameters involving different systems, the highest value should be applied, and weights should vary according to the affected system. For example, when scoring parameters 2 or 4 (systemic affections), if an animal had injuries in the respiratory, digestive, and nervous systems, the animal should receive a score of 1 (indicating 1 to 3 systems affected). Additionally, the parameter’s weight should be 3, as the nervous system is affected. Therefore, the final score would be 1 (number of systems) x 3 (lesion affecting the nervous system=highest weight) =  3.

As the body score parameter has different weights for seabirds and marine mammals, compared to sea turtles, this would cause the maximum value for these groups to be different, with 48 for the first two and 51 for the latter. To avoid confusion when comparing the HI between groups, the values were standardized to range from 0 to 1. Finally, as the HI was intended to represent an animal’s health, it was decided that 1 should represent healthy animals, and animals with all parameters at maximum values (worst condition) would have an HI of 0. To achieve this, the final value should be subtracted from 1.

The final HI should be calculated as follows:

$HI=1-\left(\frac{\sum_{i=1}^{8}Parameter_{i}}{HI_{max}}\right)$

$Parameter_{i}=Score_{i}\times Max(Weight_{i})$

 It must be noted that all parameters must be evaluated to calculate the final value. If any parameter cannot be assessed, the HI should not be determined, as missing values would artificially lower the final score.

### Comparison of HI values with a subjective health evaluation

To validate the HI under real world conditions and evaluate if a subjective evaluation based on a pathologist’s clinical experience would yield conclusions similar to those obtained through the HI calculation, 14 veterinarians from the PMP-BS teams were invited to analyze necropsy data without access to their HI values. Necropsies were selected from a first set of HI evaluations performed on carcasses collected from 2015 to 2018. Initially necropsies were sorted to include only fresh carcasses (code 2, *sensu* [19]), as these tend to be more complete. Then, they were evaluated individually and only those that were well documented and had good quality photos were kept. Finally, some cases were removed from the final set to maintain similar numbers of necropsies for the three zoological classes (seabirds, turtles, and marine mammals) and guarantee a good spread of HI values, including low (less than 0.3), intermediate (0.4 to 0.6) and high (above 0.6) HI values. This resulted in 89 specimens: 29 seabirds, 29 turtles, and 31 marine mammals.

This dataset was made available to the veterinarians, and they were asked to classify the animals’ health as “good”, “fair”, or “poor”, using histopathology reports and necropsy photos to support their assessments. To evaluate whether the Health Index difference among these three categories was significant, a Kruskal-Wallis test, with Dunn’s pairwise comparisons, was used.

It is worth noting that the selected professionals, though all working in marine tetrapod rehabilitation, were employed at different institutions and had varying levels of technical skills. This variability helped to reduce or eliminate biases from shared experiences during the evaluation.

### Assessment of the HI variability

After the veterinarian’s evaluation suggested that HI was a reliable indicator of an animals’ health (see Results), collection of data to compute HI values was included in the PMP-BS standard protocols. This allowed a broader evaluation of HI behavior and variability among and within taxons.

This broader assessment of the HI variability was done with data from carcasses necropsied by different institutions participating in the PMP-BS. Only carcasses classified as fresh (code 2) were used since they are more likely to provide all the information needed to compute the HI. In this assessment, necropsies on seabirds, marine mammals, and sea turtles, performed from August 2015 to October 2020, were included. As the HI evaluation had been included in the PMP-BS protocols, all eighty-five veterinarians of 11 institutions participating in the PMP-BS at that time were scoring the HI parameters in carcasses necropsied at their institutions. Although some parameters are subjective, using a large number of participants helps to average out these biases.

To assess the HI variability, frequency analyses were performed, to evaluate skewness and kurtosis of values. Also, the contribution of each parameter to the HI was analyzed, to evaluate if the behavior of specific parameters varied differently. For this analysis, HI values were classified into five categories (<0.20, 0.20 to 0.39, 0.40 to 0.59, 0.60 to 0.79, and 0.80 to 1.0) and values of each parameter were visually assessed among categories.

All animals were collected under permit IBAMA ABIO-640/2015

## Results

### Comparison of HI values with subjective health evaluation

**Fig 1:**
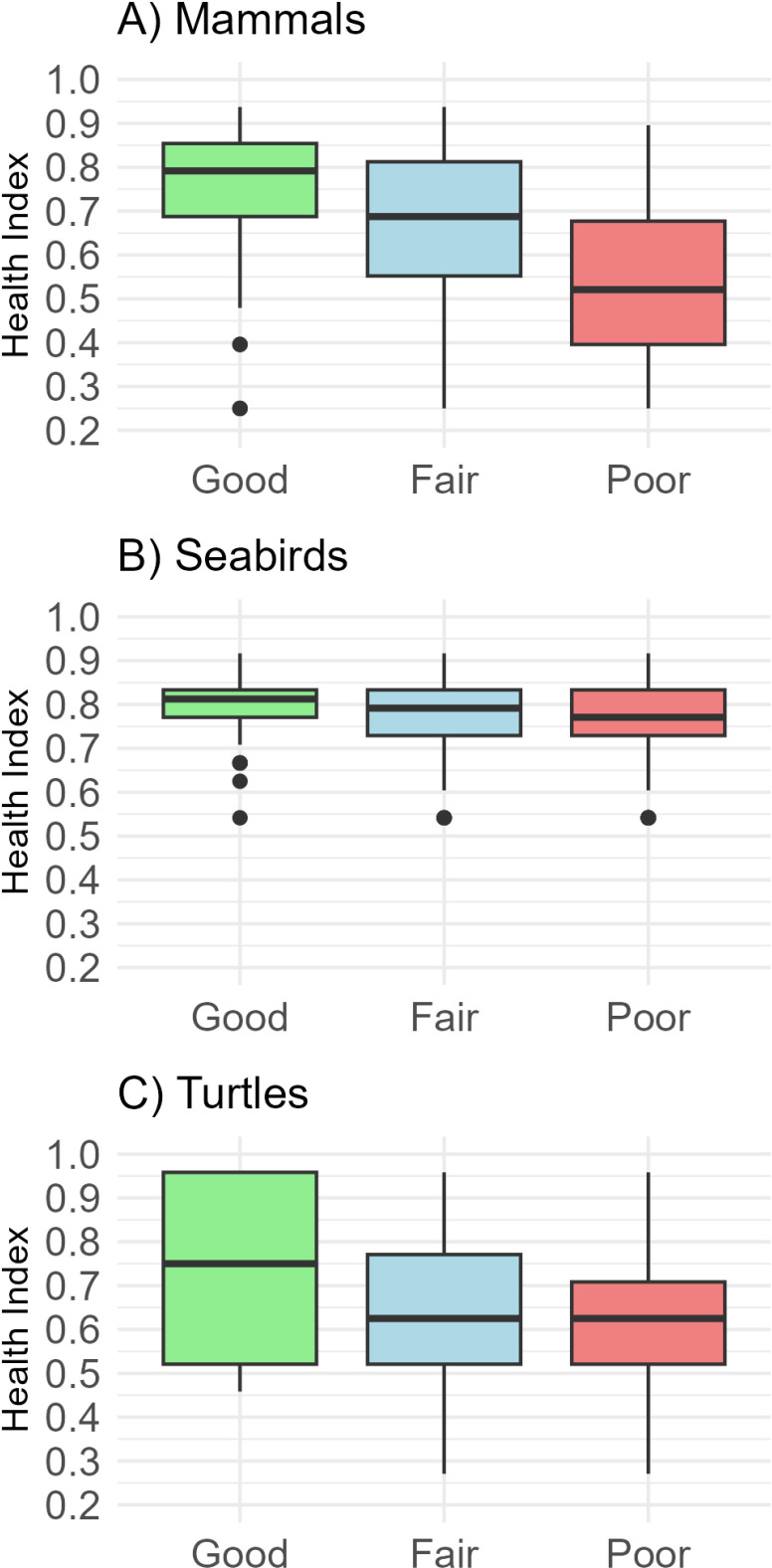
Health Index and subjective health categories. Variability in HI values (median, quartiles, range) for the subjective health categories (‘good,’ ‘fair,’ and ‘poor’) in (A) marine mammals, (B) seabirds, and (C) sea turtles.

Comparison of veterinarians’ evaluations and HI values for health categories (good, fair, and poor) showed a decreasing pattern in HI averages across categories (Fig 1). A Kruskal-Wallis test found significant differences in HI values among categories for marine mammals (H =  109.1, p <  0.0001), turtles (H =  13.29, p =  0.0013), and seabirds (H =  6.02, p =  0.048). Dunn’s *post hoc* test showed significantly lower HI values (p <  0.05) in the ‘poor’ category for marine mammals and turtles compared to ‘good’ and ‘fair.’ In seabirds, only the ‘poor’ group differed significantly from ‘good.’

### HI variability

From 2015 to 2020, the PMP-BS teams performed necropsies on 6,332 fresh specimens (code 2), establishing HIs for 54 species of seabirds, 14 species of marine mammals, and five species of sea turtles (S1 Table). Seabirds (n = 3,843) and marine mammals (n = 321) had similar HI values, with an average and standard deviation of 0.74 ± 0.10 and 0.73 ± 0.12, respectively. Sea turtles had the lowest average HI among the groups, with 0.63 ±  0.13 (n = 2168). The lowest HI found was 0.20 in a green turtle, *Chelonia mydas*. Turtles also had fewer values between 0.8 and 1.0 compared to the other groups, indicating worse health conditions. Among seabirds, the lowest HI was 0.29, observed in a kelp gull, *Larus dominicanus*, and a Magellanic penguin, *Spheniscus magellanicus*. The lowest HI for marine mammals was 0.33, recorded in a Guiana dolphin, *Sotalia guianensis*.

A summary of the HI values for the five most abundant species in each class is listed in Table 1, while the complete list of species is available in the Supporting Information. Among seabirds, the most common species was the Magellanic penguin, with 1,332 specimens presenting an average HI of 0.71 ± 0.10, the lowest average among the five most abundant seabird species. It was followed by kelp gulls and brown boobies (*Sula leucogaster*), both with an average of 0.74 ± 0.11 (Table 1).

**Table 1:** Variability of Health Index (HI) values. Average, standard deviation, minimum, maximum HI values for the most abundant species collected by the Santos Basin Beach Monitoring Project (PMP-BS) from 2015 to 2020. “Other species” indicates values for all other species except those mentioned in the table.

		Health Index (HI)			
	n	Avg.	Std. Dev.	Min.	Max.
Seabirds					
*Spheniscus magellanicus*	1,332	0.71	0.10	0.29	0.98
*Larus dominicanus*	909	0.74	0.11	0.29	0.94
*Sula leucogaster*	428	0.74	0.11	0.42	0.96
*Puffinus puffinus*	426	0.77	0.11	0.44	0.98
*Fregata magnificens*	128	0.77	0.09	0.54	0.96
Other species	582	0.75	0.10	0.35	0.96
Total	3.805	0.74	0.10	0.29	0.98
Marine mammals					
*Pontoporia blainvillei*	97	0.77	0.10	0.48	0.77
*Arctocephalus australis*	93	0.75	0.10	0.42	0.75
*Sotalia guianensis*	77	0.71	0.14	0.33	0.71
*Tursiops truncatus*	10	0.69	0.11	0.46	0.69
*Stenella frontalis*	9	0.65	0.10	0.46	0.65
Other species	32	0.64	0.13	0.44	0.94
Total	318	0.73	0.12	0.33	0.98
Sea turtles					
*Chelonia mydas*	2,103	0.63	0.13	0.20	0.96
*Caretta caretta*	47	0.68	0.10	0.43	0.92
*Lepidochelys olivacea*	10	0.66	0.14	0.35	0.88
*Dermochelys coriacea*	3	0.70	0.13	0.59	0.88
*Eretmochelys imbricata*	3	0.82	0.05	0.76	0.88
Total	2,166	0.63	0.13	0.20	0.96

Of the 321 marine mammal specimens necropsied, 83.18% belonged to three species: *Pontoporia blainvillei* (n = 97), *Arctocephalus australis* (n = 93), and *Sotalia guianensis* (n = 77). Among the reptiles, green turtles (*Chelonia mydas*) represented 97.1% (2,103) of the 2,166 specimens evaluated, with the lowest average HI among the five species of turtles evaluated (HI = 0.63).

**Fig 2:**
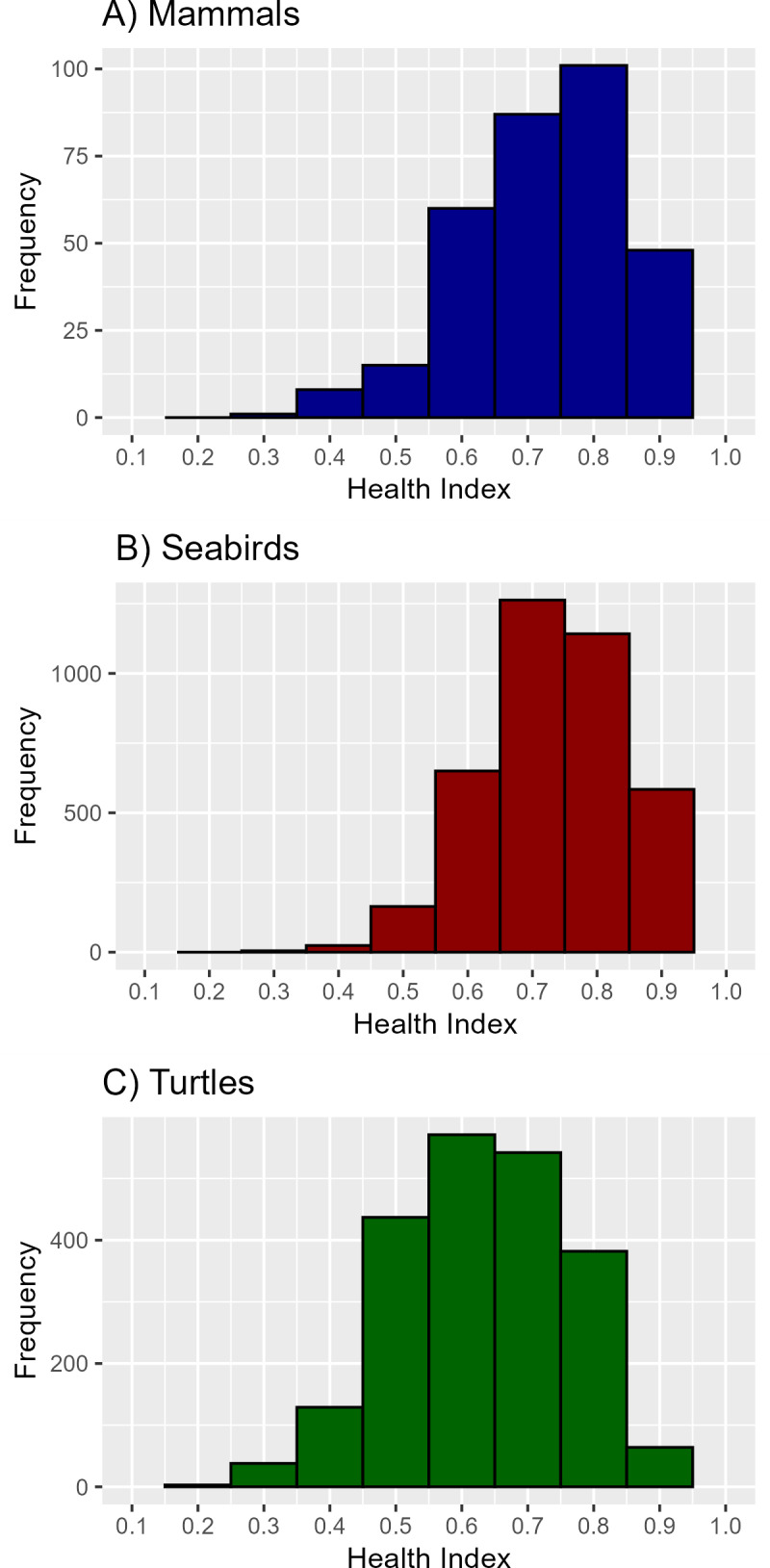
Distribution of Health Index (HI) values. Frequency of observed HI values for (A) marine mammals, (B) seabirds, and (C) sea turtles from necropsies performed by the Santos Basin Beach Monitoring Project (PMP-BS) from 2015 to 2020.

The HI varied differently among the classes. However, for all three there was a tendency towards values above 0.5. A frequency analysis indicated a slight negative skewness in HI values for seabirds, marine mammals, and sea turtles, with a Fisher-Pearson standardized moment coefficient (g1) of -0.55, -0.63, and -0.33, respectively (Fig 2).

**Fig 3:**
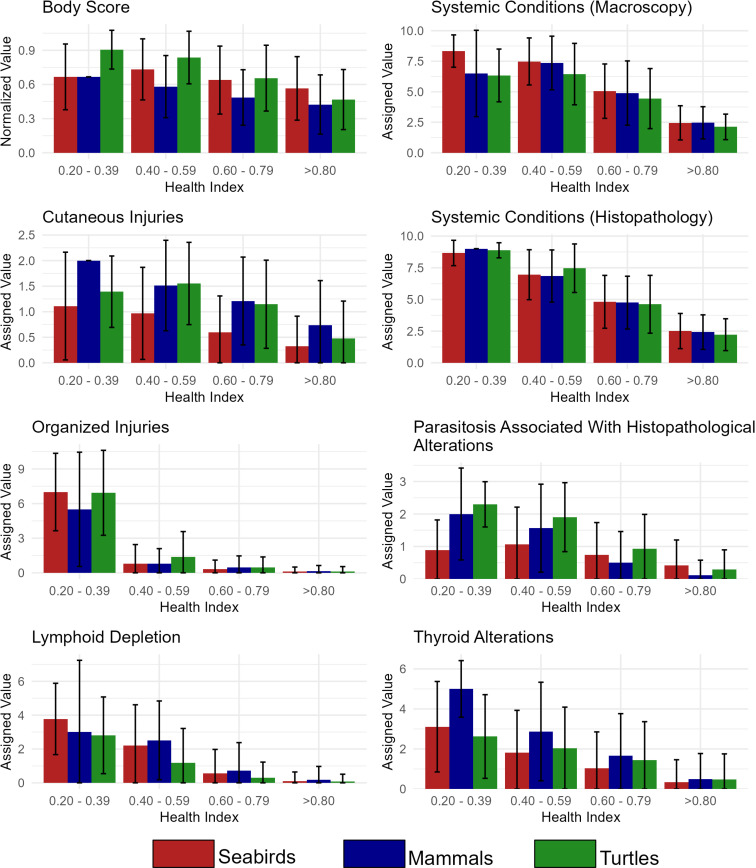
Variability in HI parameters. Distribution of means and standard deviation of values assigned to each HI parameter for each HI class. No animals had HI below 0.20. Body Score data were standardized from 0 to 1 as this is the only parameter with different weights between classes.

When the average values assigned to each parameter were compared across categories, it was possible to observe a pattern for the three classes: lower HI values, indicating worse health conditions, were associated with higher parameter scores (Fig 3). One should note that high values represent good health only when the final HI value is calculated. When individual parameters are considered, the more impaired a system, the higher the value. Overall, HI behavior is consistently represented across all parameters, with no specific parameter making a distinctive contribution to the final HI value.

## Discussion

Assessing an animal’s health is naturally challenging and laden with individual subjectivity. Pathologists seek to detect structural modifications that indicate an imbalance in the organism through physical signs capable of disrupting the proper performance of bodily functions. However, conceptualizing the state of health and disease is enormously complex, especially in veterinary medicine [20].

Thus, it was expected that there would be differences among professionals in the field regarding the interpretation of a given animal’s health conditions. While analyzing the veterinarians’ data, we noticed that some of them consistently rated animals with “poor” health, while others often rated them as “good.” We chose not to exclude these individuals from the analyses, recognizing they represent the range of users who may use the Health Index in the future. Despite these differences, the HI behaved as expected: animals categorized as in good health had higher HI values, while those in poor health had lower values. Therefore, we conclude that the HI values align with veterinarians’ assessments and accurately represent an animal’s overall health.

Although the focus of this work was an evaluation of the HI itself, our results indicate that sea turtles exhibit the poorest health conditions among the three classes in the sampled area, even though data analyzed refers mainly to one species, the green turtle. In the study area, stranded animals often have low body condition scores and a variety of health issues [21]. Additionally, they are severely affected by threats that alter their metabolic states, leading to conditions such as malnutrition, emaciation, and deficiencies in their immune status [22–24]. Various infectious and parasitic diseases may contribute to this scenario, including fibropapillomatosis, a neoplastic condition caused by chelonid herpesvirus 5 (ChHV5), with the potential to lead to immunosuppression, anemia, and increased vulnerability to secondary infections, thereby raising the parasitic load [22]. Additionally, the ingestion of anthropogenic debris, consistently reported in these species [25], not only reduces nutrient intake by creating a false sense of satiety, but also contributes to microlesion formation in the gastrointestinal mucosa. These lesions may trigger chronic inflammatory processes, worsening emaciation [24,26]. In Brazil, five species of sea turtles are present, with three (60%) classified as Vulnerable (*Lepidochelys olivacea*, *Caretta caretta*, and *Dermochelys coriacea*), one (20%) as Threatened (*Chelonia mydas*), which was the most frequently observed species in our study, and one (20%) as Critically Endangered (*Eretmochelys imbricata*) [27,28,29]. Anthropogenic interactions, such as bycatch in fishing gear, are considered the greatest threats to marine turtle populations [30,31]. A study conducted in northeastern Brazil found that 29.1% of stranded sea turtles showed evidence of interactions with human activities, with 15% specifically linked to interactions with fishing nets [24]. Low HI values indicate a debilitated health state, which may cause animals to stay longer periods at the surface, being more subject to vessel collisions and consume more marine debris, as they have difficulty in foraging.

Seabirds and marine mammals exhibited similar HI values, with average scores of 0.74 and 0.73, respectively. However, the wide variety of species within each class limits the evaluation of these results at this taxonomic level, as these species occupy different ecological niches and do not necessarily share the same resources or face the same pressures. For example, Magellanic penguins (*Spheniscus magellanicus*) exhibited the lowest health index (0.71) among seabird species that had more than thirty specimens evaluated. Annual strandings of large numbers of individuals of this species in Southern Brazil are common [32,33], as after migrating from northern Argentina, Magellanic penguins arrive extremely debilitated in areas monitored by the PMP-BS. The juveniles’ inexperience in capturing food at sea, combined with the enormous energy expenditure of migration, results in body mass loss, dehydration, anemia, and increased parasitic load [33–35]. These conditions can potentially contribute to a lower final HI value.

Among marine mammal species with more than ten evaluated specimens, franciscana dolphins (*Pontoporia blainvillei*) exhibited the highest average HI (0.77). This species, endemic to the subtropical coastal regions of Brazil, Uruguay, and Argentina, has small, fragmented populations and is considered the most endangered cetacean in Latin America [36]. Bycatch in gillnets has been identified as the primary cause of mortality in these dolphins [37]. Between 2016 and 2018, the average annual mortality reached 543 individuals, largely due to accidental captures in fishing nets. Considering the available population estimates of approximately 6,800 individuals [38], statistical modeling indicates that the current rates of bycatch are unsustainable, being almost five-fold higher than the potential biological removal estimate of 110 animals [36]. Thus, we consider that higher HI values in this case reflect acute causes of death, primarily drowning, with a lower presence of diseases.

The inherent subjectivity in each veterinarian’s health assessment underscores the importance of creating a method that objectively quantifies the alterations observed in a necropsy. Thus, the use of the HI allows for a quantitative and practical analysis of the health of individuals or groups of individuals. The data presented here constitute a baseline on the general health of stranded marine tetrapod populations in the area. Continuous monitoring of HI patterns will allow for future comparisons and the early detection of trends in the health of these animals. Differences in HI values between classes can guide conservation actions and management strategies that may vary depending on the species in question. Additionally, with a quantitative value for an animal’s health it is possible to evaluate spatial and temporal variations in health, helping to identify more subtle impacts on a species.

## Supporting information

S1 Table Health Index (HI) average and variability for seabirds, marine mammals, and turtles necropsied by the Santos Basin Beach Monitoring Project (PMP-BS) between August 2015 and October 2020.
(DOCX)

S1 Dataset Raw HI values. “SIMBA Individual Identifier” refer to the specimen identification at SIMBA database, available at http://simba.petrobras.com.br
(CSV)
