# Supplementary material for: Development of a health index for stranded marine tetrapods
Source: PLoS One. 2025 Mar 31;20(3):e0319250. doi: 10.1371/journal.pone.0319250 (PMC11957283; doi:10.1371/journal.pone.0319250)
Supplement: S1 Table — Health Index (HI) average and variability for seabirds, marine mammals, and turtles necropsied by the Santos Basin Beach Monitoring Project (PMP-BS) between August 2015 and October 2020. (DOCX) [file pone.0319250.s001.docx]

**Table S1 – Health Index (HI) values all species.** HI values (average, standard deviation, minimum, maximum) for all species of seabirds, marine mammals, and turtles necropsied by the Santos Basin Beach Monitoring Project (PMP-BS) between August 2015 and October 2020.

|  | **n** | **Average** | **Standard deviation** | **Minimum** | **Maximum** |
| --- | --- | --- | --- | --- | --- |
| **Seabirds** |  |  |  |  |  |
| *Spheniscus magellanicus* | 1332 | 0.71 | 0.10 | 0.29 | 0.98 |
| *Larus dominicanus* | 909 | 0.74 | 0.11 | 0.29 | 0.94 |
| *Sula leucogaster* | 428 | 0.74 | 0.11 | 0.42 | 0.96 |
| *Puffinus puffinus* | 426 | 0.77 | 0.11 | 0.44 | 0.98 |
| *Fregata magnificens* | 128 | 0.77 | 0.09 | 0.54 | 0.96 |
| *Phalacrocorax brasilianus* | 123 | 0.72 | 0.10 | 0.44 | 0.96 |
| *Procellaria aequinoctialis* | 54 | 0.77 | 0.10 | 0.35 | 0.94 |
| *Sterna hirundinacea* | 40 | 0.80 | 0.08 | 0.63 | 0.92 |
| *Thalasseus acuflavidus* | 38 | 0.76 | 0.10 | 0.56 | 0.92 |
| *Thalassarche melanophris* | 37 | 0.75 | 0.07 | 0.60 | 0.90 |
| *Calonectris diomedea* | 24 | 0.73 | 0.10 | 0.56 | 0.90 |
| *Macronectes giganteus* | 23 | 0.75 | 0.12 | 0.42 | 0.90 |
| *Puffinus gravis* | 23 | 0.69 | 0.11 | 0.40 | 0.88 |
| *Thalassarche chlororhynchos* | 22 | 0.77 | 0.09 | 0.56 | 0.94 |
| *Sterna hirundo* | 20 | 0.80 | 0.09 | 0.58 | 0.94 |
| *Rynchops niger* | 18 | 0.73 | 0.10 | 0.54 | 0.90 |
| *Ardea alba* | 15 | 0.75 | 0.09 | 0.63 | 0.90 |
| *Egretta thula* | 11 | 0.77 | 0.09 | 0.58 | 0.90 |
| *Himantopus melanurus* | 11 | 0.77 | 0.11 | 0.54 | 0.92 |
| *Nycticorax nycticorax* | 11 | 0.72 | 0.10 | 0.54 | 0.90 |
| *Sterna trudeaui* | 9 | 0.76 | 0.08 | 0.63 | 0.90 |
| *Thalasseus maximus* | 9 | 0.76 | 0.08 | 0.67 | 0.90 |
| *Haematopus palliatus* | 8 | 0.80 | 0.07 | 0.67 | 0.90 |
| *Ardea cocoi* | 7 | 0.73 | 0.12 | 0.52 | 0.88 |
| *Pterodroma incerta* | 7 | 0.82 | 0.07 | 0.75 | 0.92 |
| *Calonectris diomedea borealis* | 6 | 0.63 | 0.06 | 0.52 | 0.71 |
| *Calidris alba* | 5 | 0.78 | 0.08 | 0.67 | 0.88 |
| *Pterodroma mollis* | 5 | 0.78 | 0.10 | 0.65 | 0.85 |
| *Fulmarus glacialoides* | 4 | 0.67 | 0.05 | 0.60 | 0.73 |
| *Oceanites oceanicus* | 4 | 0.76 | 0.06 | 0.71 | 0.85 |
| *Anous stolidus* | 3 | 0.81 | 0.04 | 0.77 | 0.85 |
| *Butorides striata* | 3 | 0.72 | 0.04 | 0.69 | 0.77 |
| *Pachyptila desolata* | 3 | 0.67 | 0.05 | 0.60 | 0.73 |
| *Stercorarius chilensis* | 3 | 0.84 | 0.01 | 0.83 | 0.85 |
| *Stercorarius maccormicki* | 3 | 0.81 | 0.12 | 0.67 | 0.96 |
| *Stercorarius parasiticus* | 3 | 0.82 | 0.04 | 0.77 | 0.85 |
| *Calidris fuscicollis* | 2 | 0.80 | 0.05 | 0.75 | 0.85 |
| *Nyctanassa violacea* | 2 | 0.71 | 0.00 | 0.71 | 0.71 |
| *Pachyptila vittata* | 2 | 0.79 | 0.02 | 0.77 | 0.81 |
| *Pluvialis dominica* | 2 | 0.82 | 0.01 | 0.81 | 0.83 |
| *Procellaria conspicillata* | 2 | 0.78 | 0.01 | 0.77 | 0.79 |
| *Puffinus griseus* | 2 | 0.71 | 0.08 | 0.63 | 0.79 |
| *Stercorarius pomarinus* | 2 | 0.67 | 0.04 | 0.63 | 0.71 |
| *Botaurus pinnatus* | 1 | 0.65 | 0.00 | 0.65 | 0.65 |
| *Bubulcus ibis* | 1 | 0.88 | 0.00 | 0.88 | 0.88 |
| *Calidris canutus* | 1 | 0.83 | 0.00 | 0.83 | 0.83 |
| *Calidris himantopus* | 1 | 0.83 | 0.00 | 0.83 | 0.83 |
| *Charadrius semipalmatus* | 1 | 0.83 | 0.00 | 0.83 | 0.83 |
| *Daption capense* | 1 | 0.85 | 0.00 | 0.85 | 0.85 |
| *Gallinago paraguaiae* | 1 | 0.75 | 0.00 | 0.75 | 0.75 |
| *Halobaena caerulea* | 1 | 0.85 | 0.00 | 0.85 | 0.85 |
| *Macronectes halli* | 1 | 0.81 | 0.00 | 0.81 | 0.81 |
| *Onychoprion fuscatus* | 1 | 0.63 | 0.00 | 0.63 | 0.63 |
| *Phimosus infuscatus* | 1 | 0.60 | 0.00 | 0.60 | 0.60 |
| *Pluvialis squatarola* | 1 | 0.71 | 0.00 | 0.71 | 0.71 |
| *Porphyrio martinica* | 1 | 0.77 | 0.00 | 0.77 | 0.77 |
| *Stercorarius longicaudus* | 1 | 0.65 | 0.00 | 0.65 | 0.65 |
| *Tringa flavipes* | 1 | 0.83 | 0.00 | 0.83 | 0.83 |
| *Tringa melanoleuca* | 1 | 0.67 | 0.00 | 0.67 | 0.67 |
| **Marine Mammals** | **n** | **Average** | **Standard deviation** | **Minimum** | **Maximum** |
| *Pontoporia blainvillei* | 97 | 0.77 | 0.10 | 0.48 | 0.77 |
| *Arctocephalus australis* | 93 | 0.75 | 0.10 | 0.42 | 0.75 |
| *Sotalia guianensis* | 77 | 0.71 | 0.14 | 0.33 | 0.71 |
| *Tursiops truncatus* | 10 | 0.69 | 0.11 | 0.46 | 0.69 |
| *Stenella frontalis* | 9 | 0.65 | 0.10 | 0.46 | 0.65 |
| *Arctocephalus tropicalis* | 7 | 0.64 | 0.08 | 0.50 | 0.64 |
| *Steno bredanensis* | 6 | 0.58 | 0.14 | 0.46 | 0.58 |
| *Kogia breviceps* | 4 | 0.66 | 0.09 | 0.52 | 0.66 |
| *Balaenoptera acutorostrata* | 2 | 0.67 | 0.23 | 0.44 | 0.67 |
| *Lontra longicaudis* | 2 | 0.68 | 0.09 | 0.58 | 0.68 |
| *Megaptera novaeangliae* | 2 | 0.90 | 0.04 | 0.85 | 0.90 |
| *Stenella longirostris* | 2 | 0.64 | 0.07 | 0.56 | 0.64 |
| *Feresa attenuata* | 1 | 0.65 | 0.00 | 0.65 | 0.65 |
| *Globicephala macrorhynchus* | 1 | 0.63 | 0.00 | 0.63 | 0.63 |
| *Kogia sima* | 1 | 0.67 | 0.00 | 0.67 | 0.67 |
| *Lobodon carcinophaga* | 1 | 0.44 | 0.00 | 0.44 | 0.44 |
| *Otaria flavescens* | 1 | 0.63 | 0.00 | 0.63 | 0.63 |
| *Phocoena dioptrica* | 1 | 0.81 | 0.00 | 0.81 | 0.81 |
| *Stenella coeruleoalba* | 1 | 0.63 | 0.00 | 0.63 | 0.63 |
| **Turtles** | **n** | **Average** | **Standard deviation** | **Minimum** | **Maximum** |
| *Chelonia mydas* | 2103 | 0.63 | 0.13 | 0.96 | 0.20 |
| *Caretta caretta* | 47 | 0.68 | 0.10 | 0.92 | 0.43 |
| *Lepidochelys olivacea* | 10 | 0.66 | 0.14 | 0.88 | 0.35 |
| *Dermochelys coriacea* | 3 | 0.70 | 0.13 | 0.88 | 0.59 |
| *Eretmochelys imbricata* | 3 | 0.82 | 0.05 | 0.88 | 0.76 |
